# Discovery of Hyperactive Antifreeze Protein from Phylogenetically Distant Beetles Questions Its Evolutionary Origin

**DOI:** 10.3390/ijms22073637

**Published:** 2021-03-31

**Authors:** Tatsuya Arai, Akari Yamauchi, Ai Miura, Hidemasa Kondo, Yoshiyuki Nishimiya, Yuji C. Sasaki, Sakae Tsuda

**Affiliations:** 1 Bioproduction Research Institute, National Institute of Advanced Industrial Science and Technology (AIST), Sapporo 062-8517, Japan; tatarai0926@gmail.com (T.A.); a.miura@aist.go.jp (A.M.); h.kondo@aist.go.jp (H.K.); y.nishimiya@aist.go.jp (Y.N.); 2 Graduate School of Frontier Sciences, The University of Tokyo, Kashiwa 277-8561, Japan; ycsasaki@edu.k.u-tokyo.ac.jp; 3 Graduate School of Life Sciences, Hokkaido University, Sapporo 060-0810, Japan; akari.yamauchi.jp@gmail.com; 4 OPERANDO Open Innovation Laboratory, National Institute of Advanced Industrial Science and Technology (AIST), Tsukuba 305-8563, Japan

**Keywords:** antifreeze protein (AFP), thermal hysteresis (TH), stag beetle, ice binding, freeze avoidance, supercooling, tandem repeat, evolutionary origin

## Abstract

Beetle hyperactive antifreeze protein (AFP) has a unique ability to maintain a supercooling state of its body fluids, however, less is known about its origination. Here, we found that a popular stag beetle *Dorcus hopei binodulosus* (*Dhb*) synthesizes at least 6 isoforms of hyperactive AFP (*Dhb*AFP). Cold-acclimated *Dhb* larvae tolerated −5 °C chilled storage for 24 h and fully recovered after warming, suggesting that *Dhb*AFP facilitates overwintering of this beetle. A *Dhb*AFP isoform (~10 kDa) appeared to consist of 6−8 tandem repeats of a 12-residue consensus sequence (TCTxSxNCxxAx), which exhibited 3 °C of high freezing point depression and the ability of binding to an entire surface of a single ice crystal. Significantly, these properties as well as DNA sequences including the untranslated region, signal peptide region, and an AFP-encoding region of *Dhb* are highly similar to those identified for a known hyperactive AFP (*Tm*AFP) from the beetle *Tenebrio molitor* (*Tm*). Progenitor of *Dhb* and *Tm* was branched off approximately 300 million years ago, so no known evolution mechanism hardly explains the retainment of the DNA sequence for such a lo­ng divergence period. Existence of unrevealed gene transfer mechanism will be hypothesized between these two phylogenetically distant beetles to acquire this type of hyperactive AFP.

## 1. Introduction

**Figure 1:**
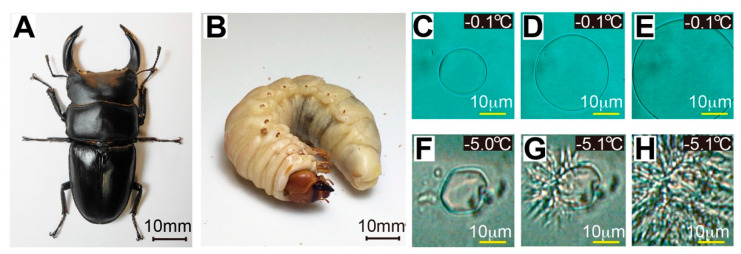
A stag beetle *Dorcus hopei binodulosus* (*Dhb*) and a bursting ice-crystal growth observed for its hemolymph at the freezing point. (**A**) An adult form of *Dhb* and (**B**) its final instar larva. The adult is uniquely equipped with a pair of antlers like a stag. (**C**) A photomicroscope image of a single ice crystal in ordinary supercooled water (−0.1 °C), which shows a rounded disk-like morphology. (**D**,**E**) The disk expanded when the temperature was held, implying that there is no antifreeze substance. (**F**) A photomicroscope image of a single ice crystal prepared in larval hemolymph, which kept unchanged even if the temperature was changed between −5.0 and 0 °C because of the ice-binding of hyperactive AFPs. The hyperactive AFP-bound ice crystal is uniquely modified into a lemon-like shape. (**G**) Bursting ice growth occurred from a tip of the lemon crystal when it further lowered the temperature to freezing point (−5.1 °C). (**H**) The bursting ice growth continues to show a vein-like pattern, which is also a known phenomenon for hyperactive AFPs.

Stag beetles include approximately 1200 species of insects belonging to the family *Lucanidae*, characterized by a pair of long horns, which resemble antlers of a stag, protruding from the mandibles of the male (Figure 1A) [1,2]. Stag beetles established immense popularity in Japan [3,4] as evidenced by cartoons, trading card games, fan websites, or the Japanese Samurai helmet of the 1500s, which was decorated with an ornament called “Kuwagata” designed after the stag beetle (Supplementary Material, Figure S1). Among them, *Dorcus hopei binodulosus* (*Dhb*) (Figure 1A,B) is especially popular because of its stout large body [5]. The *Dhb* beetle is found in northeastern China, Korea, and Japan that have an icy winter season, while it uniquely possesses a long-life overwintering nature; larvae live for 1–2 years until pupation in a rotten tree or underground, and the adult lives for 3–5 years on the ground [2,5]. Because hyperactive antifreeze protein (AFP) discovered in fishes, insects, and bacteria is known to facilitate their cold-survival [6], we speculated whether the *Dhb* beetle produces this protein in its body fluids. Since only four beetles, namely *Tenebrio molitor*, *Dendroides canadensis*, *Anatolica polita*, and *Microdera punctipennis*, are known to synthesize hyperactive AFP composed of a consensus sequence [7,8,9,10] and all of them belong to the same superfamily *Tenebrionoidea*, less is known about the origins of this protein, while the *Dhb* beetle belongs to a different superfamily *Scarabaeoidea* [2]. Therefore, if *Dhb* contains hyperactive AFP, the discovery provides a first clue to understanding the origin of this protein as well as new resources (i.e., stag beetles) to explore more new species of hyperactive AFP.

Aqueous solutions that remain in liquid phase by lowering the temperature below the melting point are called supercooled solutions, in which numerous “embryo” ice crystals are present dispersively [11,12]. When the temperature is further lowered and thermal movement of embryo ice crystals decreases, they aggregate rapidly to form a large cluster to nucleate numerous ice particles, thereby changing the water molecules into a multi-crystalline state, which is the general ice crystal structure. Note that a substance other than water molecules sometimes catalyzed the aggregation process [11]. The overwintering, cold-adapted insects have long been known for their ability to stabilize such a supercooled state of their body fluids at subzero temperatures, leading to protection from freezing [13]. The supercooled state, or semi-frozen state, is also stabilized with low molecular weight solutes in the body fluids, such as polyhydroxy alcohol (ex. glycerol), sugars (ex. treharose), amino acids (ex. proline), and/or glycolipids. Insects that equip such cold-survival mechanisms are called freeze-avoidance species, which cannot recover once their body fluids are frozen entirely. In contrast, freeze-tolerant species can recover from the freezing of their body fluids, though usually only extracellular fluids are thought to be frozen by secreting proteinaceous ice nucleators that protects inside of cells from freezing [13]. The hyperactive AFP synthesized in the freeze-avoidance insects strongly arrests the growth of embryonic single ice crystals generated in supercooled water [7]. This mechanism indeed prevents aqueous solutions from freezing down to approximately −5 °C. This protein is therefore regarded as a novel cryoprotectant to realize quality preservation of vaccines, cells, and organs around −5 °C without formation of ice blocks [14]. Note that the AFP’s ability to stabilize the supercooling state is evaluated by a difference between freezing and melting points of the AFP solution, which is called thermal hysteresis (TH) [15]. 

The hyperactive AFP, *Tm*AFP, was first identified in 1997 from the hemolymph of the beetle *T. molitor* (*Tm*), which exhibited a significant TH activity (~5 °C) [7]. *Tm*AFP is a mixture of nine isoforms (Mw = 8.4–12 kDa), consisting of 7–11 tandem repeats of a 12-residue consensus sequence, namely TCTxSxNCxxAx, where x is any amino acid residue [16]. A homologous AFP isoform (*Dc*AFP), containing the “TCT sequence,” was identified from another beetle *D. canadensis* that shares 46%–66% sequence identity with *Tm*AFP isoforms, and is composed of the same 12-residue sequence repeats with occasional insertions of additional amino acids [13]. This 12-residue sequence is not identified in either fish-, plant-, fungi-, nor bacteria-derived AFP. The spruce budworm *Choristoneura fumiferona* is not a beetle, but synthesizes AFP (SbwAFP, 9 kDa) composed of the TxT sequence (x is variable) and exhibits a high TH activity (5 °C) [17]. Extensions of TxT, such as TxTxTxT, were identified for other hyperactive AFPs from the longhorn beetle *Rhagium inquisitor* (*Ri*AFP, Mw = 12.8 kDa) [18] and the inchworm of the geometer moth *Campaea perlata* (*iw*AFP, Mw = 3.5–8.3 kDa) [19]. Hence, TxT is considered a key structural motif for significant TH activity. The AFP from snow flea *Hypogastrura harveyi Folsom* (*sf*AFP, Mw = 6.5–15.7 kDa) [20], a primitive arthropod with six legs and no wings, is an exception. It consists of a repetitive glycine-rich sequence instead of TxT, which also exhibits high TH activity (5.8 °C). 

We repeatedly bred the *Dhb* beetle for years to obtain the final (3rd) instar larvae, which were used for cold-survival experiments and detection of hyperactive AFP synthesis in their hemolymph. We found that *Dhb* larvae tolerate −5 °C-chilled preservation for 24 h and synthesize at least six isoforms of hyperactive AFP (*Dhb*AFPs), for which we performed mitochondrial DNA and amino acid sequence analysis, recombinant-protein preparation, TH activity measurement, structural modeling, and phylogenetic analysis. Significantly, all the data obtained for *Dhb*AFP exhibited significant similarity to those identified for a known hyperactive AFP from a phylogenetically distant beetle, the *T. molitor* (*Tm*AFP).

## 2. Results and Discussion

### 2.1. Hemolymph of Dorcus Hopei Binodulosus (Dhb) Exhibits Antifreeze Activity

The stag beetle examined in this study (Figure 1A) was originally named *Dorcus curvidens binodulosus* [21] which was classified based on morphology, ecology, and karyology. Re-evaluating its detailed morphology and genome-based classification based on mitochondrial DNA (mtDNA) suggested that this beetle should be named *Dorcus hopei binodulosus* [2]. This new classification received a consensus from the scientific community and has been adopted [2,22]. Here, we extracted mtDNA from an adult sample (Supplementary Material, Figure S2) and determined its 16S ribosomal RNA (16S rRNA) sequence, which comprises 981 base pairs. This sequence exhibited 99.2% identity with the 16S rRNA sequence of *Dorcus curvidens binodulosus* (GenBank accession no. AB178292.1) [21] and was therefore designated *Dorcus hopei binodulosus*.

AFP secretion was examined in 0.8 μL hemolymph extracted from the final instar larvae of *Dhb*, which were raised according to the procedures shown in Supplementary Material, Figure S3. Of these, 40 were cold-acclimated at 4 or 10 °C in the last 2 months of breeding, and 11 were non-cold-acclimated at 25 °C during the last 2 months. The hemolymph droplet was flash frozen on the Linkam 10,002 L photomicroscope stage to form an assembly of numerous single ice crystals, from which we reduced to one single ice crystal by manipulation of the stage temperature near 0 °C [23]. We then applied slow temperature lowering (−0.1 °C min^−1^) on this ice crystal, which modified it into a lemon-like morphology (Figure 1C) and caused a vein-like bursting growth (Figure 1D,E). These are typical observations for a solution containing hyperactive AFP [6]. Notably, such changes were detected for +4 and 10 °C-acclimated larvae, while not for 25 °C-incubated larvae, indicating that *Dhb* does not synthesize AFP without cold-acclimation. The temperature at which the bursting ice growth occurred was evaluated as the freezing point. The melting point of the lemon-like single ice crystal was also evaluated by increasing stage temperature, which allowed *TH* evaluation [23]. The *TH* values of the hemolymph were variable according to a development stage and a larval size, and maximal values of 1.7 ± 0.3 °C and 4.4 ± 0.2 °C were determined for +4 and 10 °C-acclimated final instar larvae, respectively. No significant *TH* value (0 °C) was detected for the hemolymph of non-cold-acclimated larvae. Graham et al. (1977) reported a detection of comparable *TH* activity (~5 °C) for the *Tm*AFP-containing hemolymph [7]. The present observations hence suggest that *Dhb* preferably synthesizes hyperactive AFP (*Dhb*AFP) by 10 °C-cold acclimation. The *T. molitor* larvae maximize *Tm*AFP synthesis when acclimated at 4 °C [24], whereas 4 °C-acclimated *Dhb* larvae exhibited poor activity. We speculate that the *Dhb* larva finds it challenging to maintain its metabolic rate at 4 °C because of its large body (~20 g) compared with the *T. molitor* larva (<0.2 g), which leads to decreasing *Dhb*AFP production at lower temperatures.

### 2.2. Dhb Larvae Tolerate−5 °C-Preservation for 24 Hours

To examine cold survivability of final instar *Dhb* larvae acclimated at 10 °C, we performed chilled preservation of *Dhb* larvae at −5 and −10 °C for 24 h. A dry plastic box (15 cm × 10 cm × 5 cm) containing naked larvae was placed in an incubator (model LTI-601SD, EYELA, Tokyo, JPN) preset at the required temperature (Supplementary Material, Figure S3). We took out the chilled vessel after the 24 h preservation period to evaluate larval recovery after warming them to room temperature (24 °C). All 10 larvae remained unfrozen by maintaining the supercooled state during the −5 °C-preservation and fully recovered after warming (Supplementary Material, Video S1). By contrast, at −10 °C, all 10 larvae froze and did not recover. We next performed the −5 °C-experiment with water droplets attached to larval skin. This droplet seeds ice crystals on the larval body and freezes them. The ice-seeding froze all 3 larvae during the 24-h preservation at −5 °C, and the frozen larvae never recovered. Note that reproducibility of these data was verified by using additional final instar larvae raised from different male-female pairs of *Dhb* captured in Osaka Japan. Asahina and Ohyama (1969) reported that larvae of some Japanese stag beetles overwinter within a small space of rotten wood, whose temperature was evaluated at −7 °C, whereas outside air was chilled to approximately −15 °C in the middle of winter [25]. They reported that beetle larvae remained unfrozen, whereas larvae of moths, namely *Arctiidae* and *Noctuidae*, froze in the same space. Although data on ambient temperatures of the *Dhb* larval habitat in the middle of winter are non-existent, the results suggest that *Dhb* tolerating up to −5 °C can be categorized as a freeze-avoiding species, where cold-protective substances of the hemolymph, including AFP, prevents freezing for cold-survival.

### 2.3. DhbAFP and Tenebrio Molitor AFP Show Significant Similarities

**Figure 2:**
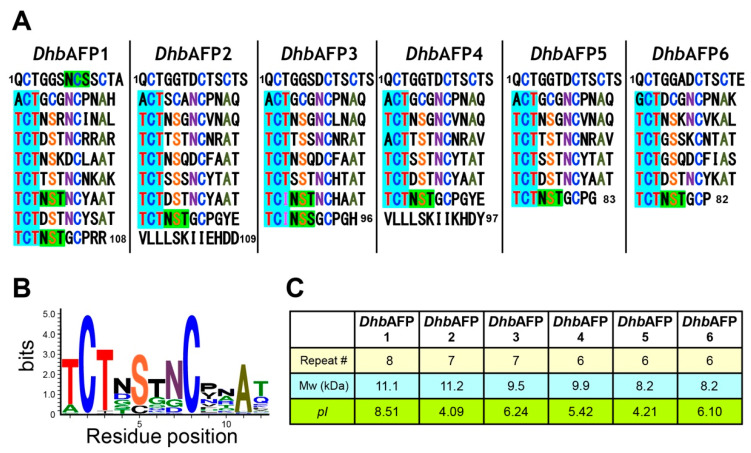
Amino acid sequence of *Dorcus hopei binodulosus* (*Dhb*)AFP. (**A**) Sequence comparison between the six *Dhb*AFP isoforms. Each consists of 6–8 tandem repeats of a 12-residue consensus sequence, in which “TCT” locating the 3 ranks of ice-like waters are highlighted with cyan. N-glycosylation sites (NxT/S, x is any residue) are highlighted with green. (**B**) The WEBLOGO plot (http://weblogo.berkeley.edu/ (accessed on 5 May 2020)) showing the consensus amino acid sequence based on the alignment of all *Dhb*AFP sequences. The 12-residue consensus sequence TCTxSxNCxxAx was deduced from this plot. (**C**) Biochemical properties of the six *Dhb*AFP isoforms. Number of tandem repeats, estimated molecular weights, and isoelectric points were evaluated for each isoform.

We constructed a cDNA library of *Dhb* according to the procedures described in Materials and Method. We synthesized primers based on the DNA sequences encoding *Tm*AFP and *Dc*AFP [8,16]. Partial sequence determination of the DNA amplified using the forward- and reverse-primers and extension of that sequence using an improved version of the primers enabled us to identify at least 6 isoforms of AFP in the final instar larva of *Dhb* (Figure 2). Alignment of the amino acid sequence with known beetle AFP isoforms is shown in Supplementary Material, Figure S4. The data revealed that *Dhb* synthesizes six isoforms of *Dhb*AFP, which are thought to exist as a mixture in the hemolymph, similar to *T. molitor* and *D. canadensis.* Many organisms, including fish, plants, and fungi, also synthesize multiple isoforms of AFP, a mix of which exhibits higher *TH* activity than any single isoform [26,27]. The first 28 residues of all *Dhb*AFP isoforms were designated as the signal peptide, similar to *Tm*AFP, suggesting that *Dhb*AFP isoforms are secreted into an extracellular space. Notably, this 28-residue signal peptide was also conserved in *Tm*AFP, but not in *Dc*AFP. The nucleotide sequence of *Dhb*AFP1 encoding the signal peptide especially shares 98.8% identity with the *Tm*AFP isoform Tq (accession no. DQ229126.1.) [28] and their amino acid sequences are 100% identical. *Dhb*AFP isoforms are composed of repetitive peptides from the 12-residue consensus sequence TCTxSxNCxxAx (x is any amino acid residue) (Figure 2B), which differentiates the number of repetitions. A BLAST search (https://blast.ncbi.nlm.nih.gov/Blast.cgi (accessed on 5 May 2020)) revealed that the top 20 polypeptides whose nucleotide sequences shared significant similarity with *Dhb*AFP isoforms were *Tm*AFP isoforms, of which *Dhb*AFP6 exhibited highest identity (90%) with the *Tm*AFP isoform Tq. DNA sequence similarity between the 6 *Dhb*AFP isoforms and 25 *Tm*AFP isoforms was 82% ± 5% on average, whereas that between the 6 *Dhb*AFP isoforms and 10 DcAFP isoforms was 67% ± 2%; *Dhb*AFP isoforms are similar to *Tm*AFP isoforms. The Blast search using DNA sequence of *Dhb*AFP3 against the *T. molitor* genome (GCA_014282415.1) further showed that 3’-untranslated region (UTR) of these two DNAs exhibited 81.9% of high sequence identity (122/149 base). The other 5’ and 3’-UTR sequences of *Dhb*AFP also exhibited 80–85% of high sequence identity with those identified in the *Tm*AFP genome. It should be noted that intron does not exist in the TmAFP genome sequence [29].

**Figure 3:**
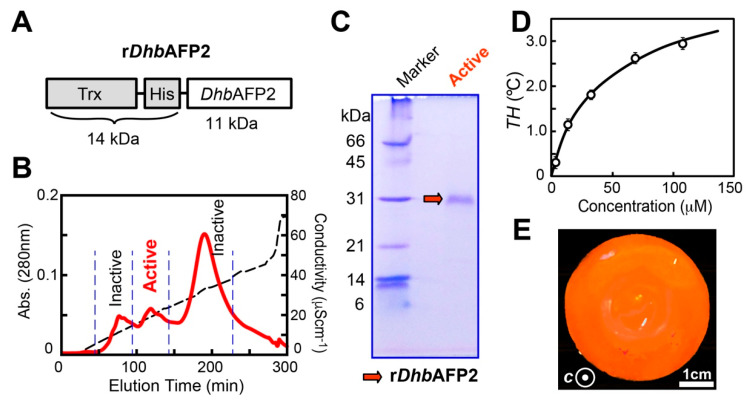
Preparation of a recombinant *Dorcus hopei binodulosus* (*Dhb*)AFP2 isoform as a fusion protein. (**A**) A schematic representation of r*Dhb*AFP2 composed of thioredoxin (Trx), histidine tag (His), and the 109-residue *Dhb*AFP2 isoform connected in tandem. (**B**) The UV absorbance (280 nm) profile of r*Dhb*AFP2 obtained with High-Q anion-exchange chromatography using an NaCl gradient (0–70 μ S cm^−1^). The lemon-like ice crystal and thermal hysteresis (*TH*) was detected for the “active” peak. (**C**) SDS-PAGE of the r*Dhb*AFP2 sample purified with Ni-column chromatography. (**D**) Concentration dependence of TH values of r*Dhb*AFP2. The measurement was performed in triplicate to draw error bars representing standard deviation. (**E**) A photograph of a single ice-crystal hemisphere, on which fluorescent (orange) r*Dhb*AFP2 adsorbs entirely to illuminate the whole surface under UV light.

The 108-residue isoform *Dhb*AFP1 is composed of eight tandem repeats of the 12-residue consensus sequence (Figure 2A). Moreover, it possesses the highest isoelectric point of all six isoforms (*pI* = 8.51, Figure 2C) due to the presence of five arginine residues, which may increase its activity with an activity enhancer [30]. *Dhb*AFP2 and *Dhb*AFP3 are composed of seven repeats of the consensus sequence. *Dhb*AFP2 is the largest isoform (11.2 kDa) and contains 109 residues. The C-terminus of *Dhb*AFP2 contains the 12-residue segment VLLLSKIIEHDD, which deviates from the repetition rule and does not exist in other AFPs. A highly similar sequence, namely VLLLSKIIKHDY, is also located in the C-terminus of the *Dhb*AFP4 isoform. The presence of this sequence in only two isoforms indicates that it does not play a crucial role in ice-binding and works as a capping structure to stabilize the tertiary fold of these isoforms. In the 6th and 7th repeat of *Dhb*AFP3, the conserved tripeptide sequence Thr-Cys-Thr (TCT) is replaced with Thr-Cys-Ile (TCI), which is not found in other *Tm*AFP isoforms. *Dhb*AFP 5 and 6 are composed of six repeats of the consensus sequence. *Dhb*AFP5 exhibited 79% similarity to the 84-residue *Tm*AFP isoform denoted 4–9 [16], which is the most abundant isoform (~50%) in *T. molitor*. The smallest isoform *Dhb*AFP6 (8.2 kDa) shares the highest similarity (80%) with another *Tm*AFP isoform denoted 2–14. Another feature of *Dhb*AFP isoforms is that the N-glycosylation site composed of the tripeptide NxT/S (x is any residue) exists within the last repeat of all *Dhb*AFP isoforms (Figure 2A, highlighted in green). More glycosylation sites exist in the N-terminal region of *Dhb*AFP1 and 6th repeat of both *Dhb*AFP1 and *Dhb*AFP3. The glycans do not cause steric interference during ice-binding in the other AFP species [16]. No significant effect of glycan on *TH* activity was verified for our recombinant isoform of *Dhb*AFP2 that contains no glycan, as it was prepared with *Escherichia coli* expression system (Figure 3).

### 2.4. A DhbAFP Isoform Equips the Properties of Hyperactive AFP 

The production of a small protein (<10 kDa) is often difficult; however, tagging it with thioredoxin (Trx) sometimes improved yield, since the resultant fusion protein tends to become more stable and soluble [31]. Here, recombinant *Dhb*AFP2 (r*Dhb*AFP2) was prepared as a 26 kDa fusion protein with a Trx-tag (Figure 3A). The “His-tag,” consisting of six consecutive histidine residues, was inserted between Trx and *Dhb*AFP2. The His-tag binds r*Dhb*AFP2 selectively to a Ni-NTA column, enabling its elution with imidazole buffer. 

A soluble fraction containing r*Dhb*AFP2 was obtained using the Ni-NTA column and dialyzed against Tris-HCl buffer (20 mM, pH 8.0) at 25 °C overnight. Notably, the solution exhibited no sign of *TH* activity before dialysis, but it was detected after dialysis (Figure 1C–E). This indicates that during dialysis, the 18 cysteines of *Dhb*AFP2 were oxidized to generate disulfide bonds, leading to a properly folded protein. Such refolding was also observed when the recombinant *Tm*AFP isoform was dialyzed [32], which depends on both the dialysis period and incubation temperature. Following dialysis, the active solution was applied to a High-Q anion-exchange column, which eluted three peaks shown in Figure 3B using an NaCl gradient (0–300 mM). The second peak exhibited *TH* activity and was applied to the Superdex 200 gel-filtration column. This gave us a fraction containing purified r*Dhb*AFP2, which was verified as a single band on 15% tricine SDS-PAGE (Figure 3C). Notably, a position mismatch on SDS-PAGE was reported for recombinant *Tm*AFP [32]. Purified r*Dhb*AFP2 exhibited hyperbolic *TH*-dependence on protein concentration, which has been reported for all AFP species [12]. A *TH* value of approximately 3 °C was evaluated at 150 µM (Figure 3D), which is consistent with that obtained for a hyperactive isoform of *Tm*AFP (3 °C at 200 µM) [32]. A single ice crystal modified into lemon-like morphology within this hysteresis gap and exhibited a vein-like bursting growth pattern, similar to those observed for the *Dhb* hemolymph (Figure 1C–E). Again, they are typical observations for hyperactive AFPs. Furthermore, the target ice plane of r*Dhb*AFP2 was examined by observing its binding onto a single ice-crystal hemisphere of a golf-ball size (ϕ = 30 mm), which was prepared by using a plastic pipe and refrigerant circulator [33]. This ice-crystal hemisphere was immersed in r*Dhb*AFP2 (0.1 mM) labeled with the fluorescent detergent tetramethylrhodamine and held at −0.8 °C for 2 h. Then, the ice hemisphere was pulled and photographed under ultraviolet (UV) light. The illumination was observed entirely on the single ice-crystal hemisphere (Figure 3E), indicating that r*Dhb*AFP2 can bind to multiple ice planes, including prism, pyramidal, and basal planes, similar to known hyperactive AFPs [33]. The r*Dhb*AFP2 was hence identified as a hyperactive AFP regardless of the Trx-tagging and removal of the glycosylation ability.

**Figure 4:**
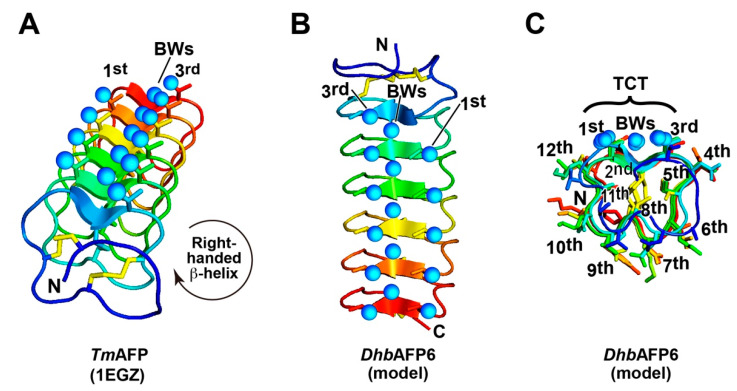
A model structure of *Dorcus hopei binodulosus* (*Dhb*)AFP6. (**A**) The X-ray crystal structure of an insect AFP from the beetle *Tenebrio molitor* (*Tm*AFP, PDB code = 1EZG) determined by Graham et al. [7]. Tandem repeats of TCT-containing sequence locate 3 ranks of oxygen atoms on side-chains T^1st^ and T^3rd^ as well as surface-bound waters (BWs) trapped in a trough. (**B**) A structural model of the *Dhb*AFP6 isoform constructed using the *Tm*AFP structure as a template. *Dhb*AFP6 and *Tm*AFP share 80% sequence identity. (**C**) A structural view down the model of *Dhb*AFP6 from N- to C-terminus. The inner core is assumed to form six disulfide bonds between C^2nd^ and C^8th^ and organize the 3 ranks of oxygen atoms on the TCT sequence.

Davies et al. determined a 1.4-Å resolution crystal structure of *Tm*AFP isoform 2–14 (1EZG.pdb) that appeared to form an extremely regular right-handed β-helix (Figure 4A) [34]. Each coil consists of the 12-residue consensus sequence T^1st^CTxSxNCxxAx^12th^, of which side-chain OH-groups of T^1st^ and T^3rd^ are aligned with another string of waters trapped in a trough, leading to the construction of three ranks of oxygen atoms along the β-helical axis [35]. This enabled us to construct the model structure of *Dhb*AFP6 (Figure 4B,C) because it shares 80% sequence similarity with *Tm*AFP isoform 2–14 [16] (Supplementary Material, Figure S4). By using the structural coordinates of *Tm*AFP (PDB ID: 1EZG) as a template, the model structure of this *Dhb*AFP6 isoform was constructed using the software MODELLER (http://salilab.org/modeller/ (accessed on 5 May 2020), Univ of California, San Francisco, CA, USA). PYMOL and CHIMERA (http://www.cgl.ucsf.edu/chimera/ (accessed on 5 May 2020)) were also used for visualizing and drawing the model. As shown in Figure 4B,C, a rounded rectangular-shaped molecule composed of 6-turns right-handed β-helices was readily modeled for *Dhb*AFP6, on which the location of the 3 ranks of oxygen atoms were speculated to be similar to *Tm*AFP, though their atom positions were not finely postulated. Formation of the 8 disulfide bonds (C^8^−C^18^, C^15^−C^21^, C^27^−C^33^, C^39^−C^45^, C^51^−C^57^, C^63^−C^69^, and C^75^−C^81^), which are thought to stabilize-helical formation were also assumed for *Dhb*AFP6 (Figure 4B,C), on which sidechains of the consensus sequence are thought to be oriented toward “out^1st^-in-out-out-in-out-out-in-out-out-in-out^12th^” directions (Figure 4C). Among them, inner pointing residues C^2nd^, S^5th^, C^8th^, and A^11th^ comprised a core region together with six disulfide bonds between C^2nd^ and C^8th^. The two-dimensional array of oxygen atoms on *Tm*AFP (Figure 4A) exhibited a perfect position match to the water’s oxygen atoms, constructing multiple ice-crystal planes [34], which can also be speculated for *Dhb*AFP6. These structural features were adopted for the larger *Dhb*AFP isoforms (Figure 2), as the *Tm*AFP structure is artificially elongated by the addition of more coils. Marshall et al. (2004) observed *TH* activity for a series of artificially prepared β-helical AFPs consisting of 6–11 tandem repeats and reported a 10–100-fold gain in activity upon going from 6 to 9 repeats [36]. The activity however decreased for 10 and 11 repeats, ascribed to imperfections of the position match between the ice-binding site and ice lattice, which occurs upon addition of too many coils.

### 2.5. Unrevealed Gene Transfer Mechanism May Exist between Dhb and Tm

**Figure 5:**
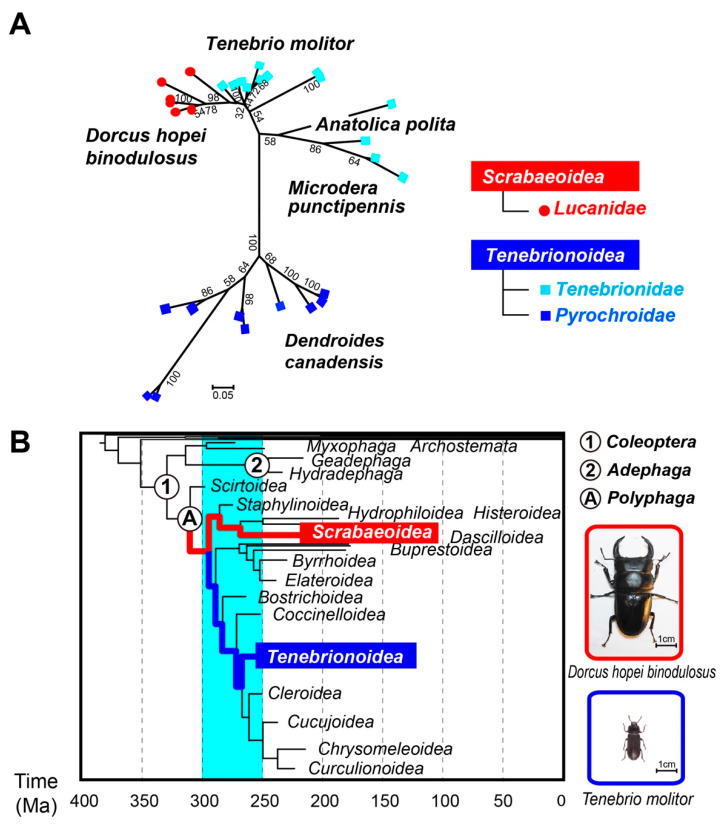
Phylogenetic relationship between the beetles synthesizing the hyperactive AFP composed of the consensus sequence TCTxSxNCxxAx. (**A**) Maximum likelihood phylogram showing a hypothetical relationship among the beetles based on analysis of a mRNA sequence alignment. The tree was created with MEGA7 software (http://www.megasoftware.net/ (accessed on 5 May 2020)) based on the HKY + G model. It used 29 mRNA sequences of beetle hyperactive AFP isoforms whose NCBI accession codes are AB264317.1–AB264322.1 (*D. h. binodulosus*), AF1591144.1–AF160497.1 (*T. molitor*), GU358703.1–GU358704.1 (*A. polita*), AY821792.1–AY821793.1 (*M. puctipennis*), and AF179408.1–AF179416.1 (*D. canadensis*). The bootstrap values for 500 replications (<30 are not shown) was shown at the nodes. (**B**) The fossil-calibration-based phylogenetic relationship between beetle superfamilies presented in Emmanuel, F. A. et al. (2017) [37]. The *T. molitor*, *D. canadensis*, *A. polita*, and *M. punctipennis* [7,8,9,10] belong to *Tenebrionoidea*. The ones examined in this study, namely *D. h. binodulosus* (*Dhb*) and *D. r. rectus* (*Drr*), belong to the phylogenetically distant superfamily *Scrabaeoidea*. Note that another beetle consisting of extensions of the TxT sequence, *Rhagium inquisitor*, belong to *Chrysomeleoidea*.

The hyperactive AFPs with the TCTxSxNCxxAx sequence have been identified for four beetle species: *T. molitor*, *D. canadensis*, *A. polita*, and *M. punctipennis* [7,8,9,10]. The present examined *Dhb* should be a new member. In addition, our preliminary results suggest that another stag beetle, *Dorcus rectus rectus* (*Drr*), synthesizes at least 14 isoforms of hyperactive AFP (Supplementary Material, Figure S5), which also exhibit the same repetitive property to *Dhb*AFP. Notably, *Dhb* and *Drr* belong to the *Scarabaeoidea* superfamily, whereas the above four beetles with the 12-residue consensus sequence belong to *Tenebrionoidea* (Figure 5). To investigate the hypothetical evolutionary relationship among these beetles, we prepared a maximum likelihood phylogenetic tree (Figure 5A) based on a total of 29 mRNA sequences of their AFP isoforms registered in the National Center for Biotechnology Information (NCBI) GenBank. In Figure 5A, *Scrabaeoidea Dhb* (6 red dots) is the closest to *Tenebriodae T. molitor* (11 cyan squares), which is consistent with the present identified similarities between *Dhb*AFP and *Tm*AFP. However, such hypothetical relationship is not supported by the most updated version of the fossil-calibration-based beetle phylogenetic tree (Figure 5B) [37]. The Figure 5B shows that superfamilies *Scarabaeoidea* and *Tenebrionoidea* belong to distant lineages, which branched off 250–300 million years ago and corresponds to the Permian glaciation period in the geological time scale [38]. One may speculate that AFPs predate speciation 300 million years ago and that most of the diverged beetles contain AFP genes. However, no *Dhb*AFP-like sequence exists in the genome of another *Scarabaeoidea* beetle, *Trypoxylus dichotomus*, for example (NCBI Genbank code: BNES01000010.1). Again, this type of hyperactive AFP was only identified for 4 beetle species in the past decades [7,8,9,10]. DNA analysis further questions the evolutionary origin of *Dhb*AFP and *Tm*AFP. Namely, the DNA sequence of *Dhb* including untranslated region, signal peptide region, and a *Dhb*AFP-encoding region exhibited a significant similarity to those identified for *Tm*AFP (Supplementary Material, Figure S4). 

Such similarity of the DNA sequences does not support the ordinary vertical evolution, since the sequences should not be retained in each superfamily for an ultimately long divergence period of approximately 300 million years. Indeed, no sign of sequence identity was detected for non-coding region of DNA in two different *Collembola* species, which were speculated to have diverged in the Permian glaciation period [39].

It has been shown that different lineages of organisms sometime synthesize similar genes through “convergent evolution”, which occurs when similar traits are acquired under selective pressure [40]. An example is the antifreeze glycoprotein (AFGP) discovered in two unrelated polar fishes, Antarctic notothenioid and Arctic cod. In Antarctic notothenioid fish, AFGP appears to be derived from a small fragment of a pancreatic trypsinogen gene, whereas in the Arctic cod, the DNA sequence is different [41,42]. Thus, the DNA sequences in two different organisms were also not retained in convergent evolution. 

A remaining possibility to explain the DNA and protein similarities between the two phylogenetically distant beetles will be an unrevealed kind of horizontal gene transfer, the movement of genetic material between unicellular and/or multicellular organisms [43]. Recent studies based on genome sequencing revealed the presence of foreign DNA sequences in the genetic material of several species of *Lepidoptra* [44]. Indeed, pro- and eukaryotic genes that moved through the horizontal gene transfer are expressed in such insect genomes [45]. Hence, it might be speculated that progenitors of *Dhb* and *T. molitor* have acquired foreign DNA encoding hyperactive AFP through unrevealed gene transfer mechanism mediated by an organism, despite Blast searches not showing any sign of hyperactive AFP synthesis in plants, fungi, nor bacteria DNA. One way to advance this study would be to discover more AFP-containing beetles and to clarify DNA sequence encoding their AFPs. Examples of the stag beetles that are popular and their breeding techniques are established include *Dorcus titanus*, *Dorcus rebrofemoratus*, *Lucanus maculifemoratus*, and *Prosopocoilus inclinatus*.

## 3. Materials and Methods

### 3.1. Construction of cDNA Library from Dhb Larva

A final instar larva of the stag beetle *Dorcus hopei binodulosus* (*Dhb*) acclimated at 10 °C was flash frozen with liquid nitrogen and homogenized with a mortar and pestle. The homogenate suspension (200 mg) was prepared using TRI reagent (2 mL, MERCK, Darmstadt, Germany) and QIAshredder (QIAGEN, Hilden, Germany). After removing the insoluble fraction from the suspension by centrifugation at 11,000× *g* for 20 min, chloroform (400 μL) was added to the supernatant to precipitate unwanted proteins and DNA. All RNAs, denoted “total RNA,” of the beetle was obtained in the aqueous phase. Total RNA was precipitated by adding 2-propanol (1 mL) and recovered with DEPC-treated water, which prevents RNA degradation. The Oligotex-dT30 mRNA purification kit (TaKaRa, Shiga, Japan) was used to extract mRNAs from total RNA. Then, reverse transcription was performed using oligo (dT) primers and obtained mRNAs for 60 min at 42 °C. This primer selectively binds the poly adenine (A) region of mRNAs, leading to the generation of single strand cDNAs (ss-cDNAs) that are complementary to mRNA sequences. The obtained ss-cDNAs were then reacted with RNase H and DNA polymerase I for 2.5 h at 16 °C to form double strand cDNAs. We utilized the ZAP-cDNA synthesis kit (TOYOBO, Osaka, Japan) for these experiments. We did not know which cDNAs encode isoforms of *Dhb*AFP at this stage. To identify them in the next step, a DNA segment called “Adaptor,” whose nucleotide sequence is mentioned in the kit, was specifically ligated to 5′- and 3′-ends of each cDNA by T4 DNA ligase overnight at 8 °C.

### 3.2. Sequence Determination of cDNA Encoding DhbAFP

The cDNA sequence that encodes an isoform of *Dhb*AFP is described as follows: (I) 5′-Adap–UTR1–Met–signal sequence–*Dhb*AFP–stop codon–UTR2–poly (A)–Adap-3′, where Adap, UTR 1 and 2, and Met denote the Adaptor, untranslated region 1 and 2, and methionine, respectively. DNA→protein translation is initiated from Met. A mature *Dhb*AFP is produced by the removal of a signal sequence located upstream (left-hand side) of the *Dhb*AFP sequence. On the basis of known DNA sequences that encode hyperactive AFPs from beetles *Tenebrio molitor* and *Dendroides canadensis* (*Tm*AFP and *Dc*AFP) [8,16], we initially prepared the following primer: (a) 5′-TGYACTGGDGSTBCYGAYTGYMVHDSKTGYAC-3′ (forward primer). This primer (a) should bind to “*Dhb*AFP” in the sequence (I), if it is a correct nucleotide sequence that can encode a protein whom cDNA exhibits high sequence similarity to that encoding *Tm*AFP and/or *Dc*AFP. We next prepared primer (b), which was designed to bind to the segment composed of “poly (A)–Adap” region in the sequence (I). (b) 5′-GAGAGAACTAGTCTCGAGTTT-3′ (reverse primer). Polymerase chain reaction (PCR) was performed with primers (a) and (b) and the cDNA library (see previous paragraph) using Ex taq DNA polymerase (TaKaRa Bio Inc, Tokyo, Japan) on our PCR instrument (GeneAmp PCR system 9700, Applied Biosystems, Foster city, USA). The following reaction cycle was used: 94 °C (1 min)–{94 °C (1 min)–50 °C (1 min)–72 °C (1 min)} × 30–72 °C (5 min)–4 °C (hold). Various cDNAs amplified with these primers, which are the promising candidates for cDNAs encoding *Dhb*AFP isoforms, were obtained using agarose gel electrophoresis (a similar example shown in Figure S3-G). The obtained cDNAs were ligated to the pGEM-T Easy vector (Promega, Madison, USA) using the Mighty TA-cloning kit (TaKaRa). The resultant plasmid, including a cDNA candidate, was then transformed into competent *Escherichia coli* JM109. Following colony PCR with universal M13RV and M13M4 primers on the agarose gel, the plasmid was collected from positive clones using the QIAprep Spin Miniprep kit (QIAGEN). We then determined their nucleotide sequence by employing BigDyeTM Terminator v3.1 cycle sequencing kit and ABI 3130xl genetic analyzer (Applied Biosystems, Foster City, CA). We found that cDNAs collected at this stage contained partial sequences, composed of half-way portion to the stop codon–UTR2 segment, but did not include the signal sequence described in (I).

On the basis of the partial sequence of cDNA candidates that encode *Dhb*AFP isoforms, we prepared the following primers: (c) 5′-TCGGGAATTCGGCACGAGG-3′ (forward primer), (d) 5′-ATAGCGGCCGCGGATCCTTAATGTCCGGGACATCCTG-3′ (reverse primer). Primer (c) was designed to bind to the upstream “5′-Adap” region in the cDNA sequence (I). Primer (d) binds to a region including the “stop codon–UTR2”. With these two primers, PCR performed using the cDNA library according to the procedures described in the previous paragraph. A problem in this experiment was that the primer (c) binds not only to “5′-Adap,” but also to the “Adap-3′” region of the sequence (I), leading to the amplification of a large number of cDNAs. Hence, the amplified DNA fragments in the size range of 500–700 bp were purified using agarose gel electrophoresis, which was the estimated size of the cDNA encoding *Dhb*AFP isoforms. Sequence analysis of our obtained samples in this time gave us revealed information, including the signal sequence, of cDNAs.

Because we could obtain information about the cDNA candidates, including the signal sequence, encoding *Dhb*AFP isoforms, we prepared the following primer: (e) 5′-GGAACATATGGCATTCAAAACGTGTGCT-3′ (forward primer). This primer was designed to bind to the signal sequence of cDNAs encoding *Dhb*AFP isoforms, whose start codon is indicated with underlined text. Hence, final PCR was performed against the cDNA library with primers (e) and (b). The original reaction cycle was slightly modified to 94 °C (1 min)–{94 °C (1 min)–56 °C (1 min)–72 °C (1 min)} × 30–72 °C (5 min)–4 °C (hold). We obtained 650 bp PCR products in this final PCR. By employing the sequence determination procedures described in the last half of the 1st paragraph of this section, we could determine at least six nucleotide sequences encoding *Dhb*AFP isoforms, which were translated into amino acid sequences shown in Figure 2 and Supplementary Material, Figure S4. 

### 3.3. Expression and Purification of rDhbAFP2 Isoform

A codon optimized DNA sequence encoding *Dhb*AFP2 isoforms (Figure 2) tagged with thioredoxin (Trx) plus six consecutive histidines (His) (r*Dhb*AFP2) was synthesized to include *Nde*I and *Not*I cleavage sites at 5′ and 3′ sites, respectively. Coexpression with the Trx-tag is known to facilitate expression of the beetle AFP from *Anatolica polita* [46]. We used this strategy to prepare r*Dhb*AFP2 in a similar manner. Histidine residues enabled affinity chromatography with the Ni-NTA column. Following the digestion of the two cleavage sites, the obtained DNA fragment encoding r*Dhb*AFP2 was inserted into the pET20b vector. This vector was then transformed into *E. coli* BL21 (DE3). Transformants selected on antibiotic plates were inoculated into 20 mL LB medium containing kanamycin and cultured at 37 °C for 16 h. Harvested cells were transferred into LB medium (1 L) containing kanamycin and cultured at 37 °C for 2−3 h. When its OD600 value reached 0.4–0.8, the expression of r*Dhb*AFP2 was induced by isopropyl-β-D-thiogalactopyranoside (0.5 mM). The obtained cells were further cultivated at 15 °C for 24 h, collected by centrifugation, resuspended in Tris-HCl buffer (0.1 M, pH 8.0) containing NaCl (0.1 M), and disrupted by sonication for 45 min. A soluble fraction of the cell lysate was obtained by centrifugation and loaded into the Ni-NTA column (QIAGEN) equilibrated with Tris-HCl (20 mM) containing NaCl (0.5 M). The r*Dhb*AFP2 was eluted by the same buffer containing 250 mM imidazole. The collected fraction was dialyzed against Tris-HCl buffer (20 mM, pH 8.0) overnight and purified using anion-exchange High-Q (Bio-Rad) column chromatography. The column-bound protein was eluted using a linear gradient of NaCl (0–300 mM). The active fraction obtained from this anion-exchange chromatography was dialyzed against and purified by gel-filtration Superdex 200 (GE-Healthcare, Amersham, UK) chromatography. The purity of the protein was confirmed by sodium-dodecyl sulfate polyacrylamide gel electrophoresis (SDS-PAGE) (Figure 3C). Purified *Dhb*AFP was dialyzed against Tris-HCl buffer (20 mM, pH 8.0) and stored at −20 °C until use.

### 3.4. Thermal Hysteresis (TH) Measurement for rDhbAFP2

The procedure to evaluate *TH* activity was described previously [26]. Briefly, hemolymph (0.8 µL) was placed in the middle of a HIRSCHMANN minicaps DE-M 18 glass capillary (ϕ = 0.92 mm) (HIRSCHMANN, Eberstadt, Germany). It was then soaked into a house-made capillary holder to set into a Linkam 10002L temperature-controlled photomicroscope stage (Linkam Science, London, UK) and observed under a Leica DMLB 100 photomicroscope system (Leica Microsystems AG, Wetzlar, Germany). The sample was flash frozen once to form a polycrystalline state of ice crystals by temperature reduction to −25 °C, and then warming to obtain a single ice crystal in that solution. Following 3-min incubation, this ice crystal was cooled again at the rate of −0.1 °C min^−1^ until bursting ice-crystal growth occurred, whose temperature was determined as the non-equilibrium freezing point [14]. The measurement was performed at least three times and averaged values were evaluated with error bars.

### 3.5. Fluorescence-Based Ice Plane Affinity (FIPA) Measurement for rDhbAFP2

The FIPA analysis was performed according the published procedures [33]. Briefly, a single ice crystal (ϕ = 3 cm) was prepared with a cylindrical mold. After determining its *c*-axis using a polarizer, a half-cut of the cylindrical ice crystal was mounted on a hollow copper tube (ϕ = 15 mm), in which −0.8 °C coolant was circulated by a refrigerant pump (Hitachi AMS-007, Hitachi, Japan). The “ice pitting method” [33] generated a six-sided star mark on the polar region of the single ice-crystal hemisphere, which indicates the *a*_1_–*a*_3_ directions of the hexagonal ice unit under atmospheric pressure [47]. The hemisphere with known orientation is then mounted onto the −0.8 ℃ chilled probe to face down the desired ice plane. Following 1–2 h incubation with a 0.1 mg/mL solution of a fluorescence-labeled AFP sample, the FIPA pattern illuminated on the ice-crystal hemisphere was observed under UV light. The fluorescence dye used was tetramethylrhodamine (5(6)-TAMRA-X, SE) (Thermo Fisher Scientific, Waltham, USA), which attaches to a lysine residue (K^103^) of the r*Dhb*AFP2 isoform.

## 4. Conclusions

The present study revealed that the popular stag beetle *Dorcus hopei binodulosus* (*Dhb*) synthesizes hyperactive AFP. Cold-acclimated *Dhb* larvae were not frozen after −5 °C-chilled preservation for 24 h and recovered after warming, thus the survival strategy of *Dhb* is freeze-avoidance. The larvae synthesize at least 6 hyperactive AFP isoforms (*Dhb*AFPs), which are tandem repeat peptides of a 12-residue consensus sequence. The *Dhb*AFPs exhibited significant similarities to a known hyperactive AFP from *T. molitor* (*Tm*AFP), where progenitors of *Dhb* and *Tm* have diverged approximately 300 million years ago. Hence, any known evolution mechanism hardly explains the retainment the DNA sequence, suggesting the existence of a recent gene transfer between these two beetles to share the hyperactive AFP. 

## Data Availability

The mRNA sequences of antifreeze protein isoforms from *Dorcus curvidens binodulosus*, which was later revised to *Dorcus hopei binodulosus*, are available at the National Center for Biotechnology Information (NCBI) GenBank (https://www.ncbi.nlm.nih.gov/genbank/ (accessed on 5 May 2020)) (Accession codes: AB264317.1–AB264322.1) and at the DNA Data bank of Japan (DDBJ) (https://www.ddbj.nig.ac.jp/ddbj/index-e.html (accessed on 5 May 2020)) (Accession codes: LC603133–LC603137). The mRNA sequences of 14 antifreeze protein isoforms from *Dorcus rectus rectus* are also available in DDBJ (Accession codes: LC598940–LC598953).

## Supplementary Materials

The following is available online at https://www.mdpi.com/article/10.3390/ijms22073637/s1.

## Abbreviations

AFP	Antifreeze protein
*Dhb*	*Dorcus hopei binodulosus*
*Tm*	*Tenebrio molitor*
TH	Thermal hysteresis
FIPA	Fluorescence-based ice plane affinity
NCBI	National Center for Biotechnology Information

## References

[1] Kim S.I., Farrel B.D. (2015). Phylogeny of world stag beetles (Coleoptera: Lucanidae) reveals a Gondwanan origin of Darwin’s stag beetle. Mol. Phylogenet. Evol..

[2] Kim S.I., Kim J.I. (2010). Review of family Lucanidae (Insecta: Coleoptera) in Korea with the description of one new species. Entomol. Res..

[3] Tournant P., Joseph L., Goka K., Couchamp F. (2012). The rarity and overexploitation paradox: Stag beetle collections in Japan. Biodivers. Conserv..

[4] Goka K., Kojima H., Okabe K. (2004). Biological invasion caused by commercialization of stag beetles in Japan. Glob. Environ. Res..

[5] Brown’s Beetles. https://abrowntks.weebly.com/dorcus-hopei.html.

[6] Scotter A.J., Marshall C.B., Graham L.A., Gilbert J.A., Garnham C.P., Davies P.L. (2006). The basis for hyperactivity of antifreeze proteins. Cryobiology.

[7] Graham L.A., Liou Y.-C., Walker V.K., Davies P.L. (1997). Hyperactive antifreeze protein from beetles. Nature.

[8] Duman J.G., Verleye N.L.-D., Goetz F.W., Wu D.W., Andorfer C.A., Benjamin T., Parmelee D.D. (1998). Molecular characterization and sequencing of antifreeze proteins from larvae of the beetle *Dendroides canadensis*. J. Comp. Physiol. B.

[9] Ma Y., Hou F., Ma J. (2009). Seasonal changes in cold tolerance of desert beetle *Anatolica polita borealis* (*Coleoptera: Tenebrionidae*) and their physiological mechanisms. Acta Entomol. Sin..

[10] Qiu L., Wang Y., Wang J., Zhang F., Ma J. (2010). Expression of biologically active recombinant antifreeze protein His-MpAFP149 from the desert beetle (*Microdera punctipennis dzungarica*) in *Escherichia coli*. Mol. Biol. Rep..

[11] Zachariassen K.E., Kristiansen E. (2000). Ice nucleation and antinucleation in nature. Cryobiology.

[12] Kristiansen E., Zachariassen K.E. (2005). The mechanism by which fish antifreeze proteins cause thermal hysteresis. Cryobiology.

[13] Duman J.G., Newton S.S., Ramløv H., Eriis D.S. (2020). Insect Antifreeze Proteins. Antifreeze Proteins.

[14] Mahatabuddin S., Tsuda S. (2018). Applications of Antifreeze proteins: Practical use of the quality products from Japanese fishes. Adv. Exp. Med. Biol..

[15] Davies P.L. (2017). Ice-binding proteins: A remarkable diversity of structures for stopping and starting ice growth. TiBS.

[16] Liou Y.-C., Thibault P., Walker V.K., Davies P.L., Graham L.A. (1999). A complex family of highly heterogeneous and internally repetitive hyperactive antifreeze proteins from the beetle *Tenebrio molitor*. Biochemistry.

[17] Graether S.P., Kuiper M.J., Gagné S.M., Walker V.K., Jia Z., Sykes B.D., Davies P.L. (2000). Helix structure and ice-binding properties of a hyperactive antifreeze protein from an insect. Nature.

[18] Kristiansen E., Ramløv H., Højrup P., Pedersen S.A., Hagen L., Zachariassen K.E. (2011). Structural characteristics of a novel antifreeze protein from the longhorn beetle *Rhagium inquisitor*. Insect Biochem. Mol. Biol..

[19] Lin F.-H., Davies P.L., Graham L.A. (2011). The Thr- and Ala-rich hyperactive antifreeze protein from Inchworm folds as a flat silk-like β-helix. Biochemistry.

[20] Graham L.A., Davies P.L. (2005). Glycine-rich antifreeze proteins from snow fleas. Science.

[21] Hosoya T., Honda M., Araya K. (2001). Genetic variation of 16S rRNA gene observed in *Ceruchus lignarius* and *Dorcus rectus rectus* (Coleoptera; Lucanidae). Entomol. Sci..

[22] Chen Y., Liu J., Cao Y., Zhou S., Wan X. (2018). Two new complete mitochondrial genomes of *Dorcus* stag beetles (*Coleoptera, Lucanidae*). Gens Genom..

[23] Bar-Dolev M., Celik Y., Wettlaufer J.S., Davies P.L., Braslavsky I. (2012). New insights into ice growth and melting modifications by antifreeze proteins. J. R. Soc. Interface.

[24] Graham L.A., Walker V.K., Davies P.L. (2000). Developmental and environmental regulation of antifreeze proteins in the mealworm beetle *Tenebrio molitor*. Eur. J. Biochem..

[25] Asahina E., Ohyama Y. (1969). Cold Resistance in insects wintering in decayed wood. Low Temp. Sci..

[26] Takamichi M., Nishimiya Y., Miura A., Tsuda S. (2007). Effect of annealing time of an ice crystal on the activity of type III antifreeze protein. FEBS J..

[27] Wang L., Duman J.G. (2005). Antifreeze proteins of the beetle *Dendroides canadensis* enhance one another’s activities. Biochemistry.

[28] Graham L.A., Qin W., Lougheed S.C., Davies P.L., Walker V.K. (2007). Evolution of hyperactive, repetitive antifreeze proteins in beetles. J. Mol. Evol..

[29] Qin W., Walker V.K. (2006). *Tenebrio molitor* antifreeze protein gene identification and regulation. Gene.

[30] Wang S., Amornwittawat N., Juwita V., Kao Y., Duman J.G., Pascal T.A., Goddard W.A., Wen X. (2009). Arginine, a key residue for the enhancing ability of an antifreeze protein of. the beetle Dendroides canadensis. Biochemistry.

[31] LaVallie E.R., Lu Z.E., Diblasio-Smith A., Collins-Racie L.A., McCoy J.M. (2000). Thioredoxin as a fusion partner for production of soluble recombinant proteins in *Escherichia coli*. Methods Enzymol..

[32] Liou Y.-C., Daley M.E., Graham L.A., Kay C.M., Walker V.K., Sykes B.D., Davies P.L. (2000). Folding and structural characterization of highly disulfide-bonded beetle antifreeze protein produced in bacteria. Protein Expr. Purif..

[33] Basu K., Garnham C.P., Nishimiya Y., Tsuda S., Braslavsky I., Davies P.L. (2014). Determining the ice-binding planes of antifreeze proteins by fluorescence-based ice plane affinity. J. Vis. Exp..

[34] Liou Y.-C., Tociij A., Davies P.L., Jia Z. (2000). Mimicry of ice structure by surface hydroxyls and water of a β-helix antifreeze protein. Nature.

[35] Graether S.P., Sykes B.D. (2004). Cold survival in freeze-intolerant insects: The structure and function of β-helical antifreeze proteins. Eur. J. Biochem..

[36] Marshall C.B., Daley M.E., Sykes B.D., Davies P.L. (2004). Enhancing the activity of a β-helical antifreeze protein by the engineered addition of Coils. Biochemistry.

[37] Emmanuel F.A., Toussaint A., Seidel M., Arriaga-Varela E., Hájek J., Král D., Sekerka L., Short A.E.Z., Fikáček M. (2017). The peril of dating beetles. Syst. Entomol..

[38] Henderson C.M., Shen S.Z., Gradstein F.M., Agterberg F.P., Gradstein F.M., Ogg J.G., Schmitz M.D., Ogg G.M. (2012). The Permian Period. The Geologic Time Scale 2020.

[39] Graham L.A., Boddington M.E., Holmstrup M., Davies P.L. (2010). Antifreeze protein complements cryoprotective dehydration in the freeze-avoiding springtail *Megaphorura arctica*. Sci. Rep..

[40] Stayton C.T. (2015). What does convergent evolution mean? The interpretation of convergence and its implications in the search for limits to evolution. Interface Focus.

[41] Chen L., DeVries A.L., Cheng C.-H.C. (1997). Evolution of antifreeze glycoprotein gene from a trypsinogen gene in Antarctic notothenioid fish. Proc. Natl. Acad. Sci. USA.

[42] Chen L., DeVries A.L., Cheng C.-H.C. (1997). Convergent evolution of antifreeze glycoproteins in Antarctic notothenioid fish and Arctic cod. Proc. Natl. Acad. Sci. USA.

[43] Keeling P.J., Palmer J.D. (2008). Horizontal gene transfer in eukaryotic evolution. Nat. Rev. Genet..

[44] Drezen J.-M., Josse T., Bézier A., Gauthier J., Huguet E., Herniou E.A. (2017). Impact of lateral transfers on the genomes of Lepidoptera. Genes.

[45] Zakharoc I.A. (2016). Horizontal Gene Transfer into the Genomes of Insects. Russ. J. Genet..

[46] Mao X., Liu Z., Ma J., Pang H., Zhang F. (2011). Characterization of a novel β-helix antifreeze protein from the desert beetle *Anatolica polita*. Cryobiology.

[47] Hobbs P.V. (1974). Appendix A Miller-Bravais indices. Ice Physics.

