# Supplementary material for: Discovery of Hyperactive Antifreeze Protein from Phylogenetically Distant Beetles Questions Its Evolutionary Origin
Source: Int J Mol Sci. 2021 Mar 31;22(7):3637. doi: 10.3390/ijms22073637 (PMC8038014; doi:10.3390/ijms22073637)
Supplement: Supplementary file 1 [file ijms-22-03637-s001.zip › Supplementary/Supplement_materials.pdf]

## Supplementary Materials for

# Phylogenetically distant beetles acquired hyperactive antifreeze protein through unrevealed gene transfer mechanism

**Tatsuya Arai, Akari Yamauchi, Ai Miura, Hidemasa Kondo, Yoshiyuki Nishimiya, Yuji C. Sasaki, and Sakae Tsuda #**

**# Correspondence author. E-mail: s.tsuda@aist.go.jp.**

**This PDF file includes Figures S1−S5.**

**Movie S1 was also saved on line indicated in the text.**

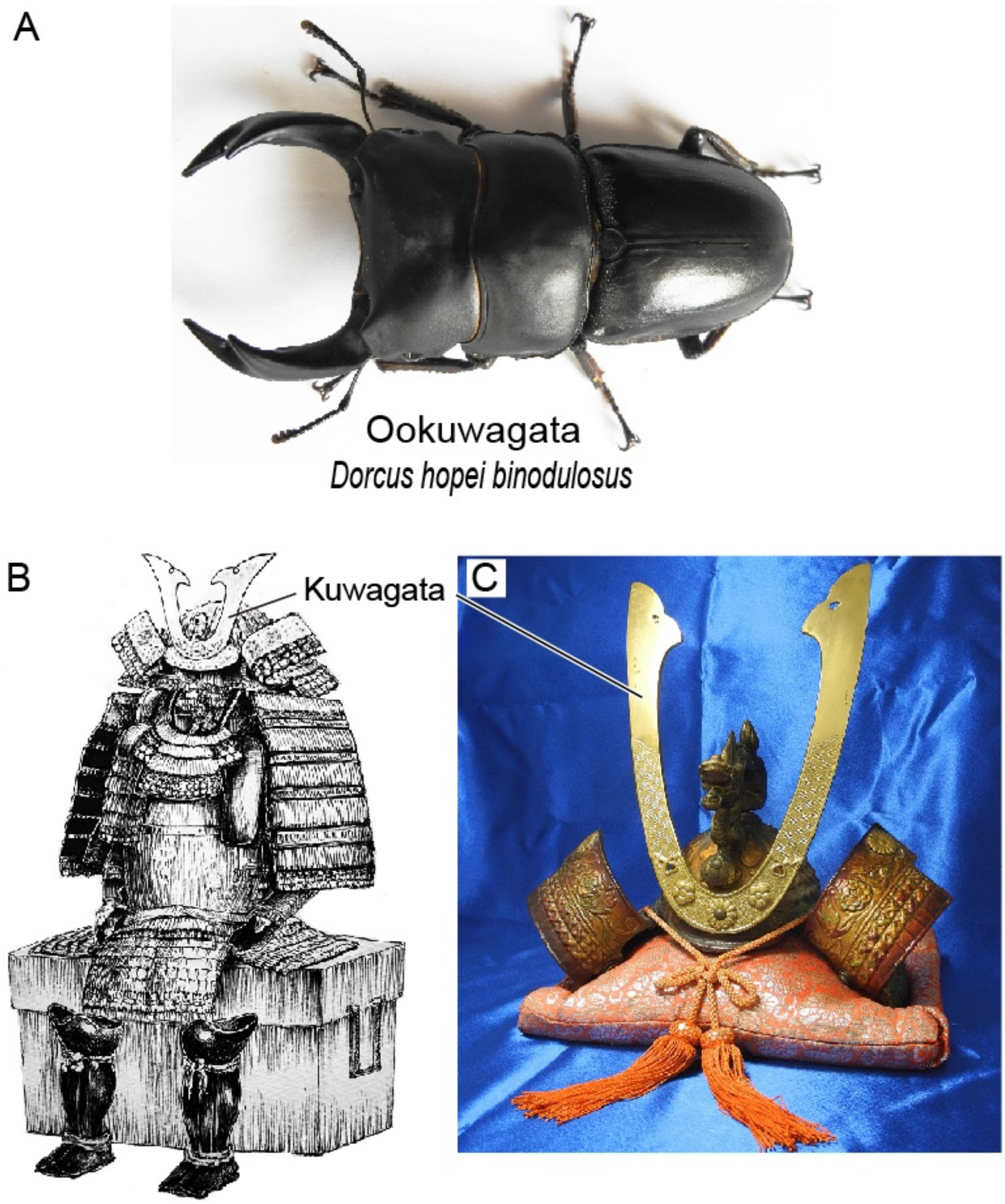


**Figure S1. Popularity of the stag beetle in Japan.** (A) A photograph of the stag beetle *Dorcus hopei binodulosus* (♂), "Ookuwagata" in Japanese, where "Oo" means giant. (B) An example of an armor protection suit for the Japanese warrior "Samurai" in the 1,500s, illustrated by Tsuda. The helmet is decorated with a pair of antlers called "Kuwagata," which was designed after the stag beetle to symbolize the toughness and power of the samurai wearing the suit. (C) A photograph of a replica of the samurai helmet decorated with Kuwagata, owned by an author (Miura).

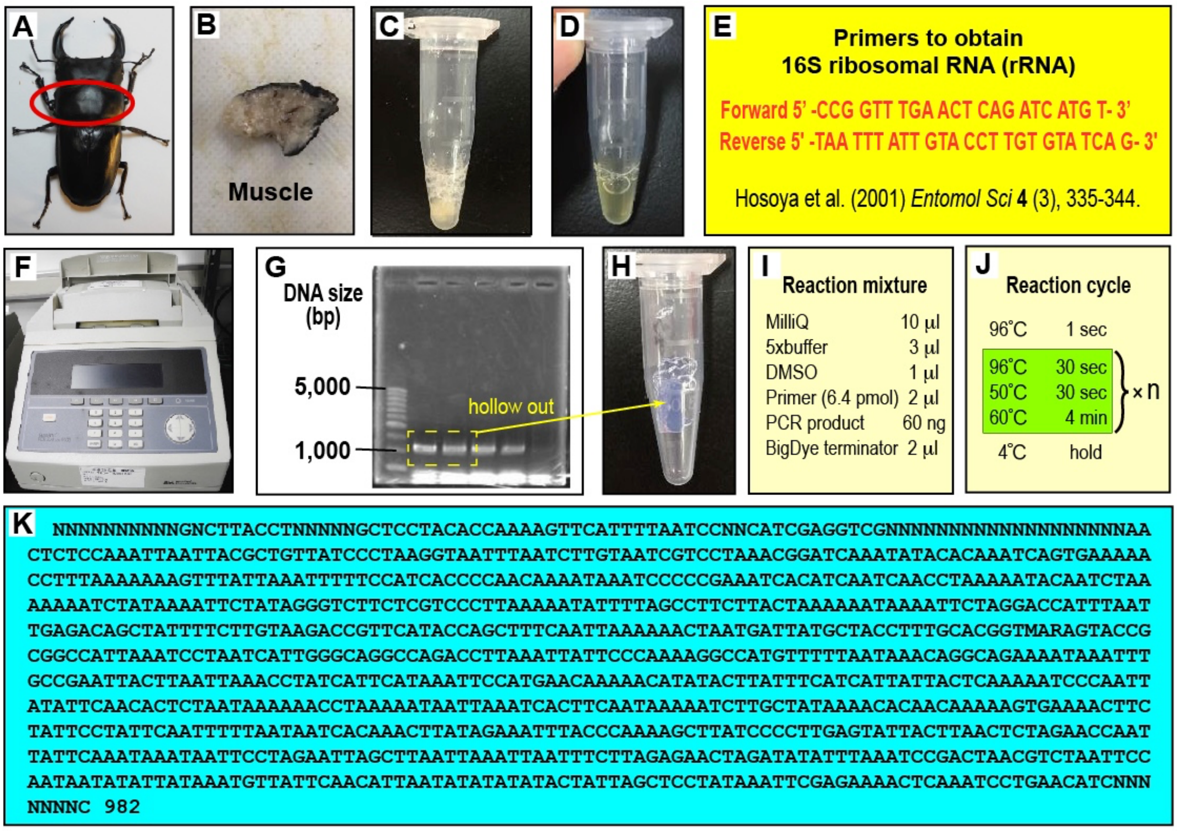


**Figure S2. Taxonomic identification of the stag beetle *Dorcus hopei binodulosus*.** (A) The adult sample (♂) to identify the DNA sequence encoding its 16S ribosomal RNA (16S rRNA). After chilling its body on ice for anesthesia, we cut out the chest (red ellipse). (B) The chest muscle (25 mg) was collected using tweezers. (C) Muscle homogenized with 200 μL lysis buffer provided in the DNeasy blood & tissue kit (QIAGEN, Hilden, Germany). Following the kit protocol, (C) was incubated with a proteinase provided by the kit at 56°C for 2 d to obtain crude extracts. (D) DNA pool obtained from the crude extract by using detergents in the kit. (E) Two primers synthesized according to Hosoya, T. *et al* [ref. 21 in the text]. to obtain DNA encoding 16S rRNA. (F) Our PCR instrument (GeneAmp PCR system 9700, Applied Biosystems, Foster city, USA). The reaction cycle of 94°C (2 min)–{94°C (40 sec)–47.3°C (50 sec)–70°C (7 min)} × 35°C–72°C (7 min)–4°C (hold) was performed using the DNA pool and primers (E) and the reaction mixture in the kit. (G) Agarose gel electrophoretogram to examine PCR products. A major band was observed at approximately 1,000 base pairs (bp), which is the estimated size for DNA encoding 16S rRNA. (H) A portion of the gel with our target DNA excised out from (G). Approximately 300 ng DNA was obtained from the gel by using QIAquick gel extraction kit 250 (QIAGEN, Hilden, Germany). (I) Reaction mixture and (J) PCR cycle to amplify our DNA. Forward and reverse PCRs were performed separately and repetitively (n = 25–50) with modifications to the program indicated in green (ex. 30 → 20 sec, 50 → 47°C). (K) The 982-bp DNA sequence encoding 16S rRNA of our beetle determined with a sequence analyzer (3500 Series Genetic Analyzer, Thermo Fisher Scientific, Waltham, USA). This final sequence, except 45 "N"s (unidentified nucleotides), shares 99.2% identity with *Dorcus curvidens binodulosus* (NCBI GenBank accession NO. AB178292.1), which was later revised to *Dorcus hopei binodulosus*.

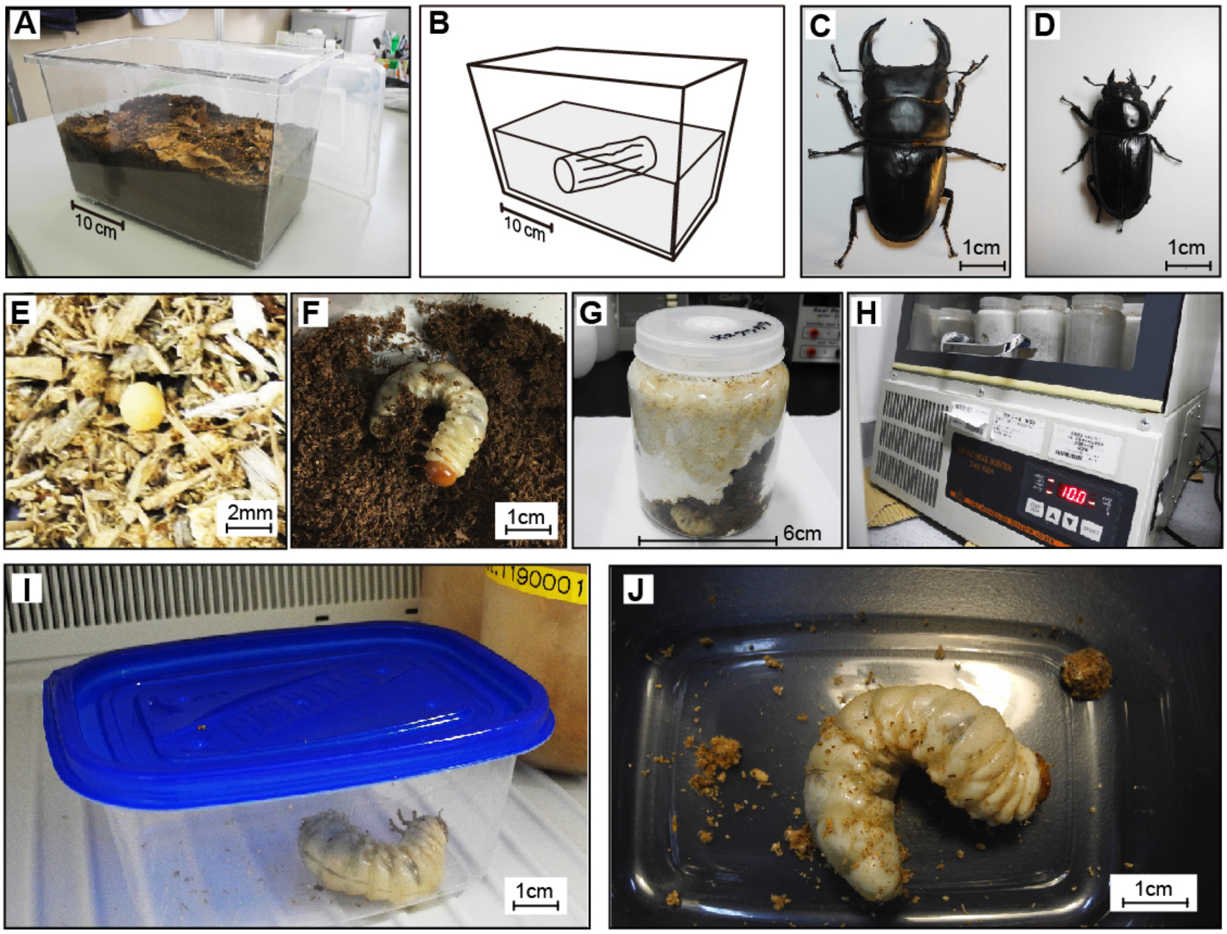


**Figure S3. Preparation of *Dorcus hopei binodulosus* larvae.** (A) An acryl case (37 × 22 × 25 cm) to breed the adult *Dhb* pair, where 1/2 the case was filled with commercial leaf mold (KBSP5.0, Four-Seasons, Sapporo, Japan). (B) A rotten piece of wood (*Quercus serrata,* $\phi$ = 12 cm, $l$ = 23 cm) was immersed into the mold. *Dhb* eggs were laid within this wood. (C) Male (♂) and (D) female (♀) *Dhb* bred to obtain larvae examined in this study. (E) A *Dhb* egg extracted from rotten wood. A total of 51 eggs were collected until the end of the summer of 2019. Each egg was separately moved into a cup (80 mL) filled with the same mold and incubated at 25°C. The infant larva ($1^{st}$ instar) hatched 1 month later. (F) The $2^{nd}$ instar larva grew to ~2.5 cm in that summer. (G) A bottle (800 mL) filled with *Quercus* sawdust, in which the larva (F) was bred until it grew to $3^{rd}$ (final) instar after incubation for 2 months at 25°C. Lignin-degraded nutrients in the sawdust are thought to accelerate larval growth. (H) A low-temperature incubator (model LTI-601SD, EYELA, Tokyo, JPN) to induce cold-acclimation of the $3^{rd}$ instar larvae. Following the 2-month incubation at 25°C, they were cold-acclimated for an additional 2 months. Forty larvae were divided into two groups for cold-acclimation at either 4°C or 10°C. The remaining eleven were bred at 25°C for reference. (I) A plastic vessel containing a 10°C-acclimated larva was placed in a freezer for 24-h chilled preservation at −5°C. (J) A *Dhb* larva, out of the freezer after 24 h of chilled preservation. A movie showing the recovery of this larva from the −5°C-chilled preservation is shown as **Video_S1.**

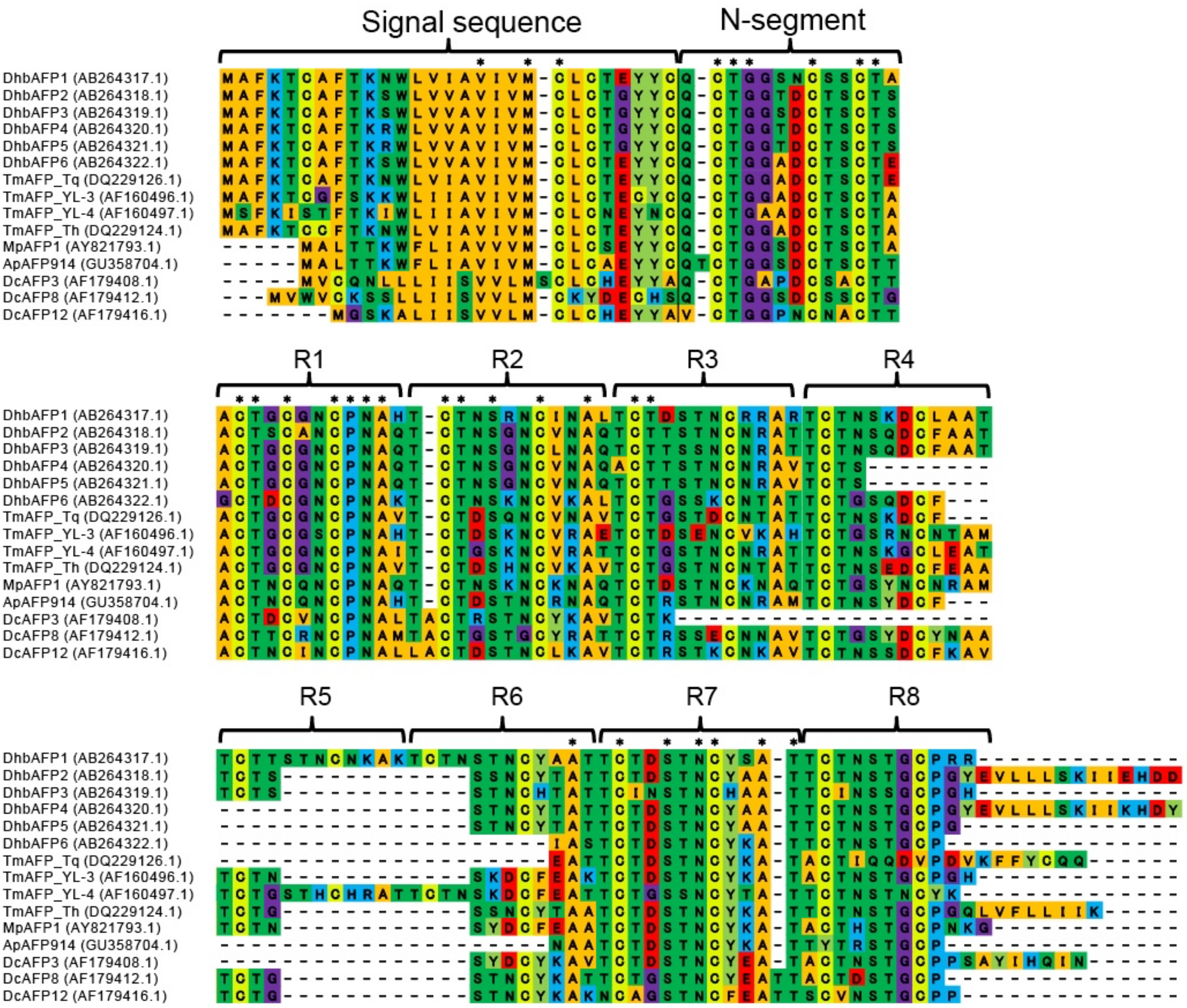


**Figure S4. Alignment of the amino acid sequence of beetle AFPs.** Amino acid sequence alignment was performed for beetle hyperactive AFPs composed of tandem repeats of the 12-residue consensus sequence TCTxSxNCxxAx using the MUSCLE algorithm in the MEGA7 software (https://www.megasoftware.net/). This figure compares the amino acid sequence of *Dorcus hopei binodulosus* (*Dhb*) AFP isoforms with those of known beetle AFP isoforms exhibiting similar repetitive sequences extracted from the NCBI database (https://www.ncbi.nlm.nih.gov/). The accession code (ex. AB264317.1) of the mRNA sequence in the NCBI GenBank (https://www.ncbi.nlm.nih.gov/genbank/) is indicated for each *Dhb*AFP isoform (ex. *Dhb*AFP1). The N-terminal segment (N-segment) of all *Dhb*AFP isoforms starts with QCT·· located next to the signal peptide sequence (signal sequence) and then tandem repeats of the 12-residue consensus sequence (R1−R8). The residues marked with an asterisk (*) are perfectly conserved in all AFP isoforms. Color indications for amino acids are as follows; neutral: green, positively charged: cyan, negatively charged: red, hydrophobic: orange, Cys: yellow, Gly: purple, and Tyr: light-green.

*Drr*AFP1 (LC598940)
$Q^{1}$CTGSPDCTSCTTACTDCGNCPNAQTCTNSQNCVSAQTCTNSGNCVNAQTCTASTNCNRAT
TCTSSKDCFAAATCTTSTNCYTAATCTDSTNCYAATACTNSSGCPNPSIKFF 113

*Drr*AFP2 (LC598941)
$Q^{1}$CTGSPDCTSCTTACTDCGNCPNAQTCTNSQNCVSAQTCTNSGNCVNAQTCTASTNCNRAT
TCTSSKDCFAAATCTTSTNCYTATTCTDSTNCYAATACTNSSGCPNPSIKFF 113

*Drr*AFP3 (LC598942)
$Q^{1}$CTGSPDCTSCTTACTDCGNCPNAQTCTNSQNCVSAQTCTNSGDCVNAQTCTASTNCNRAT
TCTSSKDCFAAATCTTSTNCYTAATCTDSTNCYAATACTNSSGCPNPSIKFF 113

*Drr*AFP4 (LC598943)
$Q^{1}$CTGGSDCSRCTAACTGCGNCPNAHTCTNSRNCINALTCTDSTNCRRARTCTSSKDCLAAT
TCTTSTNCNRAKTCTNSTNCYAARTCTNSTNCYSATTCTNSTGCPRR 108

*Drr*AFP5 (LC598944)
$Q^{1}$CTGGADCTSCTSTCTGCANCPNAQTCTNSGNCVNAQ------------ACTASRNCNKAT
TCTNSQDCFAATSCTTSTNCYTATTCINSTNCHAATTCINSSGCPGS 96

*Drr*AFP6 (LC598945)
$Q^{1}$CTGGADCTSCTSTCTGCANCPNAQTCTNSGNCVNAQ------------ACTASRNCNKAT
TCANSQDCFAATSCTTSTNCYTATTCINSTNCHAATTCINSSGCPGS 96

*Drr*AFP7 (LC598946)
$Q^{1}$CTGGADCTSCTSTCTGCANCPNAQTCTNSGNCVNAQ------------ACTASRNCNKAT
TCTKSQDCFAATSCTTSTNCYTATTCINSTNCHAATTCINSSGCPGS 96

*Drr*AFP8 (LC598947)
$Q^{1}$CTGGPDCTSCTAACTGCGNCPNAQTCTNSENCINAQ------------TCTSSTNCNRAT
TCTSSKDCFAATTCTSSTNCYTATTCTDSTNCYAATTCTNSTGCPNPSIKFV 101

*Drr*AFP9 (LC598948)
$Q^{1}$CTGGTDCTSCTVACTGCGNCPNAVTCTNSGNCVNAV------------TCTASTNCNRAT
TCTTSKDCFEAVTCTGSTNCYKATTCTDSTNCYGATTACTNSTGCPGT 96

*Drr*AFP10 (LC598949)
$Q^{1}$CTGGTDCTSCTAACTGCGNCPNAQTCTNSGNCINAV------------TCTTSTNCNRAT
TCTSSTNCYIAA------------TCTDSTNCYAATACTNSTGCPGYQVLFL 89

*Drr*AFP11 (LC598950)
$Q^{1}$CTGGTDCTSCTAACTGCGNCPNAVTCTNSGNCINAV------------TCTTSTNCNRAT
TCTSSTNCYIAA------------TCTDSTNCYAATACTNSTGCPGYQVLFL 89

*Drr*AFP12 (LC598951)
$Q^{1}$CTGGSDCTSCTVACTGCGNCPNAVTCTNSGNCINAV------------TCTSSTNCNKAT
TCTSSKDCFAAT------------TCTDSTNCYAATTCTNSSGCPGS 84

*Drr*AFP13 (LC598952)
$Q^{1}$CTGGSNCSSCTAACTGCGNCPNTHTCTNSRNCVSAR------------TCTGSTNCKKAR
TCTGSTNCYAARTCTNSTNCYSAT------------TCTNSTGCPRH 84

*Drr*AFP14 (LC598953)
$Q^{1}$CTGGSNCSSCTAACTSCGYCPNAHTCTNSRNCGNAR------------TCTSSTNCKKAR
TCTSSTNCYAAKTCTSSTNCYSAT------------TCINSTGCPRH 84

**Figure S5. Preliminary determination of primary sequences of hyperactive AFP isoforms of the stag beetle *Dorcus rectus rectus (Drr)*.** All 14 isoforms of hyperactive AFP of *Drr* (*Drr*AFP) 1–14 contain 6–8 tandem repeats of the 12-residue consensus sequence TCTxSxNCxxAx, similar to *Dorcus hope binodulosus*. The accession code (ex. LC598940) of the mRNA sequence in DDBJ (DNA Data bank of Japan) (https://www.ddbj.nig.ac.jp/ddbj/index-e.html) is indicated for each *Drr*AFP isoform (ex. *Drr*AFP1). Determination of amino acid sequence was performed according to the procedures described in Methods (pages 8–10), where the forward primer (e) was modified to 5′-GGAACATATGGCGTTCAAAACGTGTGCT-3′ to determine the *Drr*AFP sequence. The lemon-shaped ice crystal and its vein-like bursting crystal growth pattern (Figure 1 in the main text) was also observed for the hemolymph of this beetle, indicating that AFP isoforms listed in this figure exhibit a strong antifreeze activity similar to *Dorcus hopei binodulosus (Dhb) AFP* isoforms.
